# Age of Onset and Dominance in the Choice of Subject Anaphoric Devices: Comparing Natives and Near-Natives of Two Null-Subject Languages

**DOI:** 10.3389/fpsyg.2018.02729

**Published:** 2019-01-10

**Authors:** Elisa Di Domenico, Ioli Baroncini

**Affiliations:** ^1^Dipartimento di Scienze Umane e Sociali, Università per Stranieri di Perugia, Perugia, Italy; ^2^Scuola Superiore di Dottorato e di Specializzazione, Università per Stranieri di Siena, Siena, Italy

**Keywords:** age of onset, dominance, Italian, Greek, overt subject pronouns, null subject pronouns (*pro*), natives, near-natives

## Abstract

Several studies have highlighted the role of cross-linguistic influence in determining the over-use of overt subject pronouns in near-native speakers of a null-subject language as Italian. In this work we inquire on the role of factors different from cross-linguistic influence in the choice of anaphoric devices in near-natives, such as age of onset of exposure and dominance. In order to do so, comparing the productions of two groups of natives speakers, we first single out two null-subject languages, Italian and Greek, which do not differ significantly as far as subject anaphoric devices are concerned and thus instantiate a suitable language combination to investigate the role of factors other than cross-linguistic influence in bilingual speakers of these two languages (Study 1). In Study 2, we compare the productions of a group of native speakers and two groups of near-native speakers in Italian: Greek-Italian bilinguals from birth and L2ers of Italian with Greek as an L1. Results reveal that over-use of overt pronouns in near-natives occurs in the absence of cross-linguistic influence and that age of onset of exposure is a relevant factor: while bilinguals from birth do not differ from native speakers, L2ers over-use overt pronouns compared to both native speakers and bilinguals from birth. In order to establish whether dominance is a possible factor determining bilinguals’ choice of subject anaphoric devices, in Study 3, we compare two groups of Greek-Italian bilinguals from birth: bilinguals living in Greece (whose predominant language is Greek) and bilinguals living in Italy (whose predominant language is Italian). Results reveal no effect of dominance in the production of overt subject pronouns. We found, however, an unexpected effect in the predominant language of one group: bilinguals living in Greece produce significantly more null pronouns and less lexical DPs in Greek compared to bilinguals living in Italy. We interpret this effect as stemming from the need to differentiate the two languages that these bilingual speakers have to handle in everyday life. Interestingly, this effect is found in the predominant language rather than in the non-predominant one.

## Introduction

Some languages of the world are null-subject languages. In these languages the subject of finite clauses (whether matrix or embedded) can be left unpronounced, as in (1.b/d), (2.b/d) and (3.b/d):

(1) a. Gianni ha parlato
  G. spoke
b. *pro* Ha parlato
  He spoke
c. Lui ha parlato
  He spoke
d. Gianni ha detto che *pro* ha parlato
  G. said that he spoke                        Italian
(2) a. Juan habló
b. *pro* habló
c. Él habló
d. Juan dijo que *pro* habló              Spanish
(3) a. O Janis milise/ Milise o Janis
b. *pro* Milise
c. Aftos milise
d. O Janis ipe oti *pro* milise              Greek^1^

Though phonetically unrealized, the null subject is syntactically active, and is standardly indicated as *pro*, as shown in the .b and .d examples above.^2^ Given that null-subject languages have both overt (as shown in the c. examples above) and null subject pronouns, an interesting question is what the division of labor is between the two series of pronouns.

Calabrese (1986) for instance has noted that in Italian, in cases like (4), the null pronoun takes the antecedent in subject position, while the overt pronoun preferentially takes an antecedent which is not the subject:

(4) a. Quando Carlo_i_ ha picchiato Antonio_j_ *pro*_i/^∗^j_ era ubriaco
b. Quando Carlo_i_ ha picchiato Antonio_j_ lui_j/^∗^i_ era ubriaco When C. hit A *pro*/he was drunk

Noting that a post-verbal subject cannot be the antecedent of a pronoun (whether null or overt, as shown in (5)) and that *pro* can co-refer with the dative PP of so called *Psych*-verbs in preverbal position (6), the author proposes that the property ‘subject’ is not sufficient to characterize the referential properties of *pro*:

(5) a. ^∗^Ha parlato Carlo_í_ quando *pro*_ì_ è arrivato
b. ^∗^ Ha parlato Carlo_í_ quando lui_i_ è arrivato.
  Spoke C. when *pro*/he arrived
(6) Poiché a Giovanni_i_ piace Maria, *pro*_i_ fa di tutto per farsi bello ai suoi occhi
  Because G. likes M. he does everything to show off for her

Calabrese (1986) proposes that the relevant property is instead ‘Subject of primary predication’ (or Thema).^3^

As far as overt pronouns (‘stressed’ in his terms) are concerned, Calabrese (1986) assumes that they are only used when the occurrence of their referent is not expected, proposing a principle like (7):

(7) Assign the feature [+ stressed] to a pronominal X only when the occurrence of the referent of X is not expected [Calabrese, 1986: 7, ex. (18)]

Assuming that expectedness (i.e., high probability of occurrence) is correlated to low content of information, while unexpectedness (i.e., low probability of occurrence) is correlated to high content of information, he argues that (7) simply prevents giving more information than is required, and hence is a direct consequence of the second maxim of quantity of Grice (1975).^4^ ^5^

We may thus easily derive from (7) the fact that overt pronouns, at least in Italian and Greek, are required only in case of topic shift or focalization, i.e., when their referent is unexpected. But when the referent is expected, overt pronouns are impossible:

(8) a. Poiché *pro*_i_ ha visto quel film, Mario_ì_ si è spaventato
b. ^∗^Poiché lui_i_ ha visto quel film, Mario_i_ si è spaventato Because *pro*/he saw that film, M. was frightened [Calabrese, 1986 ex. (19) and (23)]
(9) a. Epidi *pro*_i_ ide ekini tin tenia, o Marios_i_ tromaxe.
b. ^∗^Epidi aftos_i_ ide ekini tin tenia, o Marios_i_ tromaxe.^6^

Things appear to work in part differently for near-native speakers, as brought to light by a number of studies. While a natural reply to (10.A) would be (10.B1) for a native speaker, near-natives may also produce (10.B2):

(10) A. Perché Giorgio si è licenziato?
  Why did G. resign
B1. Perché *pro* non sopportava più il direttore
B2. Perché lui non sopportava più il direttore
  Because *pro*/he could not stand the boss anymore [Adapted from Sorace, 2006: 507]

Tsimpli et al. (2004) for instance studied the production and comprehension of overt and null subject pronouns by native speakers of Italian and native speakers of Greek who were near-native speakers of English as an L2 and had a minimum of 6 years of residence in Britain. They were hence experiencing attrition from the L2.^7^ As for the Italian experimental subjects, the authors found a significant difference between the control and the experimental group in the choice of the matrix subject as a possible referent of the overt pronoun in the embedded sentence.^8^

Sorace and Filiaci (2006) studied the comprehension of null and overt subject pronouns in Italian by English speakers who had learned Italian as adults, reaching a near-native level of proficiency. Compared to native speakers, near-natives had a significantly higher preference for the subject of the matrix clause as a possible antecedent of overt subject pronouns.^9^

Belletti et al. (2007) were also concerned with near-native speakers of Italian whose native language was English, and who had started learning Italian as adults. Their findings on pronoun comprehension and production matched: overt pronouns were over-produced and also interpreted in co-reference with a topical antecedent by these near-native speakers.

Serratrice et al. (2004) studied the productions of an Italian-English bilingual child, finding an overuse of overt pronouns in her Italian.^10^

Taken together these studies support the idea that the over-use and over-interpretation of overt pronouns is due to cross-linguistic influence from English, a language which has only overt pronouns. But then the question is why the influence goes only from English to Italian and not in the other direction. One possibility is that these speakers chose the option compatible with both their languages: coherently with Hulk and Müller’s (2000) hypothesis, cross-linguistic influence does not occur in young bilinguals unless input from one of the languages can be analyzed through the grammar of the other language. Another possibility is, however, that overt pronouns are, for some reason, ‘simpler’ for speakers of more than one language: if so, they should be over-produced (and over-interpreted) also in the absence of cross-linguistic influence.

Sorace and Filiaci (2006: 345) quote production data collected by Bini (1993) from low-intermediate Spanish learners of Italian who use overt pronouns in contexts in which both Italian and Spanish would require a null pronoun: since cross-linguistic influence cannot be implicated in this case, the authors suggest that overt pronouns may be a default form.

Sorace et al. (2009) compare the preferences toward null and overt subject pronouns in Italian, in a [+Topic Shift] and [-Topic Shift] condition by different groups of subjects: Italian monolingual adults, Italian monolingual children, English-Italian bilingual children (6–7 and 8–10 years old, living in Italy and living in the United Kingdom), Spanish-Italian bilingual children.

In the [-TS] condition younger children chose significantly more overt pronouns than older children and adults, and older children more than adults. Children with English as the community language were more likely to choose inappropriate overt pronouns than children with Italian as the community language at the age of 6–7, but not at 8–10. Italian monolingual children aged 6–7 chose significantly more overt pronouns than adults. Spanish-Italian bilinguals were significantly more likely to opt for an overt pronoun than the monolinguals, but they were not significantly different from the English-Italian bilinguals. In the [+TS] condition bilingual children (regardless of the language combination) accepted more null subject pronouns than monolingual children.^11^

These results are very important in that they show that establishing the appropriate conditions for pronoun resolution is a phenomenon which is acquired late, in part independently from cross-linguistic influence in bilingual children, since Spanish-Italian bilingual children behaved differently from Italian monolingual children. These results also show that the pattern is not completely asymmetric, given some variability in the acceptance of null pronouns in [+TS] contexts.

The fact that the preferences of Spanish-Italian bilingual children may not be due to cross-linguistic influence has been challenged, however, by a self-paced reading study on Spanish and Italian (Filiaci et al., 2013) that found that pronominal preferences may not be the same in Italian and Spanish, although they are both null-subject languages. Sentences containing an overt pronoun congruent with a complement antecedent (as in (11)) were read significantly faster in Italian, but not in Spanish, suggesting that overt pronouns in Spanish are also compatible with a topic antecedent:

(11) a. Dopo che Giovanni_i_ ha criticato Bruno_j_ così ingiustamente, lui_j_ si è sentito offeso
b. Despues de que Bernardo_i_ criticó a Carlos_j_ tan injustamente, él_j_ se sintió muy ofendido.
  After that G./B._i_ has criticized B./C._j_ so unjustly, he_j_ felt offended

This makes the authors explicitly claim that the findings in Sorace et al. (2009) concerning the preference differences of Spanish-Italian bilingual children compared to Italian monolingual children could indeed be due to cross-linguistic influence from Spanish (Filiaci et al., 2013: 17).

This suggests that in order to verify whether the over-use/ over-acceptance of overt subject pronouns in bilinguals is not only due to cross-linguistic influence, care must be put in the choice of the language combination of bilingual speakers, since not all null-subject languages are alike in this respect.

In this work we present three studies concerning adult narrative productions in Italian and Greek by two groups of native speakers (Italian natives and Greek natives), two groups of adult Italian-Greek bilinguals from birth (Bilinguals living in Greece and Bilinguals living in Italy) and a group of adult native speakers of Greek who started to learn Italian in adulthood reaching a near-nativeness level of proficiency (L2ers).

In Study 1, we compare the productions of the two groups of native speakers, highlighting that there are no significant quantitative differences in Greek and Italian as far as the implementation of null pronouns, overt pronouns and lexical DPs are concerned, so that Italian and Greek appear as a suitable language combination to study the factors influencing bilinguals’ choices of anaphoric devices, in the absence of effects related to cross-linguistic.

In Study 2, we compare the productions in Italian of a group of native speakers and two groups of near-native speakers: bilinguals from birth and L2ers. Results reveal that near-natives over-use overt pronouns also when cross-linguistic influence is absent and that age of onset of exposure to Italian is a relevant factor in this respect: while bilinguals from birth do not differ from native speakers, L2ers over-use overt pronouns compared to both native speakers and bilinguals from birth.

In order to establish whether dominance is a possible factor determining speakers’ choice of anaphoric devices, in Study 3, we compare two groups of bilinguals: bilinguals living in Greece and bilinguals living in Italy. Results reveal no effect of dominance with respect to the production of overt-pronouns, neither in Italian nor in Greek. We found, however, an unexpected effect in the predominant language of one of the groups: bilinguals living in Greece produce significantly more null pronouns and less lexical DPs in Greek compared to bilinguals living in Italy. We interpret this effect as stemming from the need to differentiate the two languages that this bilingual group has to handle in everyday life. Interestingly, this effect is found in the predominant language rather than in the non-predominant one, and does not concern overt pronouns.

## Study 1: Subject Anaphoric Devices in Italian Natives and Greek Natives

The study conducted by Filiaci et al. (2013) reviewed in the previous section suggests that an analogous null/overt pronouns division of labor among null-subject languages should not be taken for granted. Spanish, as the authors show, differs from Italian in that overt pronouns appear to retrieve a subject antecedent to a greater extent in Spanish compared to Italian. In Study 1, we therefore compare the productions of two groups of native speakers (Italian native speakers and Greek native speakers) in order to see whether the proportion of null and overt pronouns and lexical DPs produced is comparable in the two groups. If this analysis reveals no significant differences, differences in the productions of speakers of the two languages could not be attributed to cross-linguistic influence.

### Subjects

20 subjects participated in Study 1: 10 native speakers of Italian and 10 native speakers of Greek.

Italian Natives (6 male; 4 female) had a mean age of 32 (range 19–58). They were born in Italy and had been living there by the end of testing. Three of them had a university degree, while seven had a high school degree and were attending university.

Greek Natives (4 male; 6 female) had a mean age of 29 (range 19–58). They were born in Greece and had been living there by the end of testing. Four of them had a university degree, while six had a high school degree and were attending university.

### Materials and Methods

#### Ethical Considerations

There is no ethical committee in our institutions, and for this reason this study could not undergo an ethical reviewing process, not required according to the guidelines of our institution and national regulations in such cases. The subjects in this study were adults who participated in it on a voluntary basis and came to the place of data collection for this purpose only. They were informed about the general aims of the research and gave their written informed consent to the treatment of the data they produced, including the publication of the results. In order to protect their anonymity, subjects were coded only by progressive numbers in the data analysis.

#### Procedure

Subjects were asked to watch a short movie (The Pear Film) and then tell the story.^12^ Subjects productions were recorded and then transcribed with the help of the CLAN system (part of the CHILDES tools, MacWhinney, 2000). Subjects were tested individually in a quiet room and the interviewer did not interact with them during their narration.

#### Defining the Reference Total

The narrations collected with the procedure described above were then analyzed in order to study the occurrences of null and overt subject pronouns as well as of subject lexical DPs chosen by the speakers. Given the nature of the task (semi-spontaneous production), the two corpora contained a great variety of clausal types. Not all of them, however, can be considered suitable environments to study speakers’ choice of subject referring expression, since in many of these clausal types no true clause-internal choice is possible as far as their subject is concerned, since it is syntactically determined. For instance, this is the case in subject relatives, where, according to a raising analysis of this clausal type, the subject is the copy of the moved head of the relative, or in pseudo-relatives, where the antecedent must be overt and the internal subject is invariably null. In subject clefts the subject is focalized, hence it cannot be null. As for absolute gerundive and participial, adjectival and prepositional small clauses, their subject is standardly assumed to be PRO. The subject is also syntactically determined in Italian infinitives (whether control, raising or ACC-ing) and in Greek *na* and *ke* clauses, when they are complement of certain verbs.^13^ Finally, the subject of existential sentences is syntactically determined, in Italian as well as in Greek.^14^

For this reason, we kept in what we call the ‘Reference Total’ only those clausal types whose subject can be chosen clause-internally by the speaker, i.e., finite and copular sentences as well as non-subject relatives and non-subject clefts.

Since we adopted this ‘free clause-internal choice’ criterion, other cases had to be excluded, as well.

Finite sentences whose subject was the narrator or included narrator+ interviewer were also excluded, since they were in the first person (singular or plural), and a choice between a null pronoun, an overt pronoun or a lexical DP is only possible in the third person, lexical DPs being excluded from first and second person.^15^

Some of the sentences were used to introduce (rather than to resume) a Discourse Referent, and since first mention is always lexical, we excluded those sentences as well.

In this way we obtained the Reference Total, which consists of 387 sentences produced by the Italian natives and 454 sentences produced by the Greek natives. In this Reference Total we analyzed the occurrences of null pronouns, overt pronouns and lexical DPs.

### Results^16^

Null subject pronouns are the most employed anaphoric device (67.18% by Italian natives; 69.38% by Greek natives), followed by lexical DPs (24.28% by Italian natives; 23.12% by Greek natives), while overt pronouns are quite rare (6.20% in the Italian natives Reference Total; 3.37% in the Greek natives Reference Total). We have singled out another resumption device which we call ‘other’ and which consists of various quantificational expressions such as It. ‘*uno*’ (one), ‘*uno dei tre*’ (lit. one out of the three), ‘*tutti*’ (all of them), Gr. ‘*enas apo aftous’* (one of them). Instanced of ‘other’ are quite rare, as well (2.06 % in the Reference Total of the Italian natives; 3.74% in the Reference Total of the Greek natives).

**FIGURE 1:**
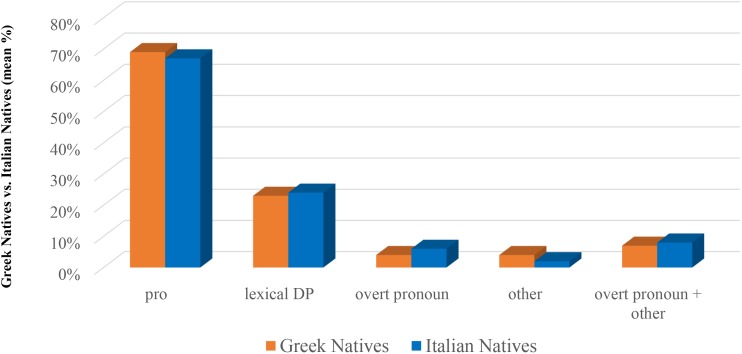
Subject anaphoric devices in Greek Natives and Italian Natives.

A χ^2^-test reveals no significant difference between the two groups neither for *pro* (χ^2^ = 0.4675, n.s.) nor for lexical DPs (χ^2^ = 0.1561, n.s.), overt pronouns (χ^2^ = 2.2157 with Yates correction, n.s.), ‘other’ (χ^2^ = 1.4977 with Yates correction, n.s.). The same goes for the case of collapsing overt pronouns and ‘other’ (χ^2^ = 0.0844 with Yates correction, n.s.). Figure 1 reports the comparisons.^17^

### Discussion

Results show a very similar pattern characterizing Italian native speakers’ and Greek native speakers’ choice of referring expressions. In particular, they show that there are no significant differences in the amounts of the various referring expressions chosen by the speakers. Null pronouns are widely employed, followed by lexical DPs, while overt pronouns are quite rare in both groups. Results are important in that they show that Italian and Greek, despite their differences, are comparable languages, at least as far as production is concerned, with respect to the relative amount of anaphoric devices employed.^18^ This in turn means that in bilingual speakers of both these languages, no effect related to cross-linguistic influence is expected with respect to the issue at stake. With this in mind, we move to Study 2.

## Study 2: Subject Anaphoric Devices in Native and Near-Native Speakers of Italian

The results of Study 1 show that native speakers of Italian and native speakers of Greek do not differ significantly in the production of null and overt pronominal as well as lexical DP subjects. Thus, we do not expect any effects of cross-linguistic influence with respect to the anaphoric devices chosen by the speakers of both these languages. These data will be relevant to establish whether the over-use of overt pronouns observed in near-natives by the studies described in the Introduction is due to cross-linguistic influence alone, or whether other factors are involved as well: if Greek-Italian bilingual speakers over-use overt pronouns, this cannot be due to cross-linguistic influence.

### Subjects

30 subjects participated in Study 2: the group of 10 native speakers of Italian of Study 1 (henceforth Natives), a group of 10 Greek-Italian bilinguals from birth living in Greece (henceforth Bilinguals in Greece), and a group of 10 native speakers of Greek who started to learn Italian after puberty and had reached a near-native level of proficiency in this language (henceforth L2ers).

Natives have been described in the section ‘Subjects’ of Study 1. As for Bilinguals in Greece (3 male; 7 female) their mean age at the time of testing was 21 (range 16–33). They were living in Greece at the time of testing and had been living there most of their lives. They were tested in Greece. They were all bilinguals from birth, with one parent native speaker of Greek and one parent native speaker of Italian. Despite living in Greece, they all also used Italian on a regular basis.^19^ As for their education, 6 of them were attending the last year of the Italian State School of Athens, 1 had just graduated from this school, 3 had a university degree, and had previously attended the Italian State School of Athens.

As for L2ers (4 male; 6 female), their mean age at the time of testing was 32 (range 21–52). They were born in Greece and had spent there at least the first 18 years of their lives. At the time of testing they were living in Italy, where they were tested. The length of their residence in Italy was 7 years on average Their age of onset of exposure to Italian ranged from 15 to 28. As for their education 4 had a university degree and 6 had a high school degree and were attending university in Italy.

### Materials and Methods

#### Ethical Considerations

The same ethical considerations holding for Study 1 (see section “Ethical Considerations”) hold for this study as well. The data collection at the Italian State School of Athens (which concerns 6 subjects, see section “Subjects” above) was authorized by the school pro-Rector.

#### Procedure

As described for Study 1, subjects were asked to watch The Pear Film and then tell the story, first in Italian and then in Greek. The subjects productions were recorded and then transcribed with the help of the CLAN system. Subjects were tested individually in a quiet room and the interviewer did not interact with them during their narration.

#### The Near-Nativeness Level of the Subjects

In order to see whether the materials collected were appropriate for our study, we first performed a near-nativeness test on these materials, adapting White and Genesee’s (1996) near-nativeness test along the lines of Contemori et al. (2015) and Dal Pozzo and Matteini (2015). Three native speakers of Italian evaluated the oral productions in Italian of the experimental subjects, indicating their judgments with respect to five distinct aspects (morphology, syntax, vocabulary, pronunciation, fluency) on a scale of 10 cm.^20^ The mean value of these five judgements constitutes the near-nativeness value assigned by each judge to each participant. The final near-nativeness value of each participant corresponds to the mean value of the values expressed by each judge. A speaker is considered near-native if her/his mean value ranges from 8.5 to 9.5.

Taken as a group, Bilinguals in Greece had a mean value of 8.98 (range 8.70–9.28). L2ers had a mean value of 8.88 (range 8.50–9.33). In order to have a line of comparison for our study, we had the same three judges evaluate the Natives productions as well: taken as a group, Natives had a mean value of 9.79 (range 9.64–9.96).

Although not entirely relevant for this study (but see section “Extension” below), we also asked three native speakers of Greek to evaluate the productions of the Bilinguals and the L2ers in Greek.^21^ Taken as a group, Bilinguals had a mean value of 9.34 (range 8.61–9.80) while L2ers had a mean value of 9.73 (9.56–9.92). Note that the same Greek judges evaluated the productions of the group of the Greek native speakers of Study 1. Taken as a group, they had a mean value of 9.87 (range 9.75–10).^22^

#### Defining the Reference Total

The Reference Total was derived with the same procedure described for Study 1. As mentioned, the Natives’ Reference Total consists of 387 sentences. The Bilinguals in Greece Reference Total consists of 241 sentences, while the L2ers’ Reference Total consists of 255 sentences.

### Results^23^

As in Study 1, *pro* is the preferred anaphoric device in all groups (67.18% Natives, 63.90% Bilinguals, 60.68% L2ers), followed by lexical DPs (24.28% Natives, 29.46% Bilinguals, 23.52% L2ers), overt pronouns (6.20% Natives, 5.80% Bilinguals, 14.50% L2ers) and ‘other’ (2.06% Natives, 0.82% Bilinguals, 1.17% L2ers).

As for *pro*, Natives do not differ from Bilinguals (χ^2^ = 0.7126, n.s.) nor from L2ers (χ^2^ = 2.7540, n.s.); Bilinguals and L2ers do not differ from each-other (χ^2^ = 0.5122, n.s.).

Lexical DPs as well appear equally employed: Natives do not differ from Bilinguals (χ^2^ = 2.0502, n.s.) nor from L2ers (χ^2^ = 0.0487, n.s.), Bilinguals and L2ers do not differ from each-other (χ^2^ = 2.2426, n.s.).

Similarly, as to the category ‘other,’ Natives do not differ from Bilinguals (χ^2^ = 0.7688 with Yates correction, n.s.) nor from L2ers (χ^2^ = 0.2918 with Yates correction, n.s.), Bilinguals and L2ers do not differ from each-other (χ^2^ = 0.0040 with Yates correction, n.s.).

Things appear different as far as overt pronouns are concerned. Natives do not differ from Bilinguals (χ^2^ = 0.0008 with Yates correction, n.s.) but they significantly differ from L2ers (χ^2^ = 11.3923 with Yates correction, significant at *p* < 0.05; 0.01; 0.005). L2ers also significantly differ from Bilinguals (χ^2^ = 9.2462 with Yates correction, significant at *p* < 0.05; 0.01; 0.005).

**FIGURE 2:**
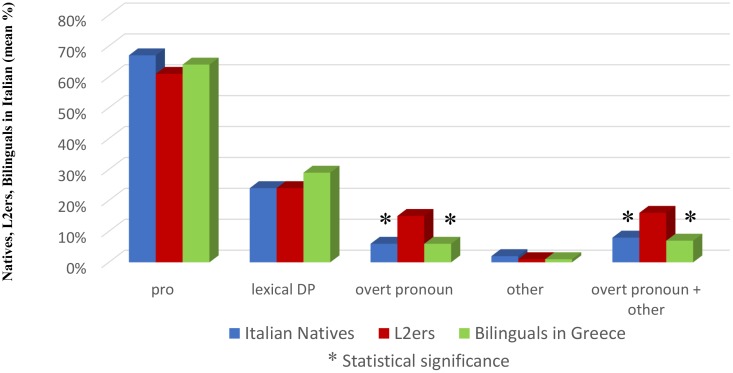
Subject anaphoric devices in Italian: Natives, L2ers and Bilinguals.

These differences are replicated when overt pronouns and ‘other’ are collapsed: Natives do not differ from Bilinguals (χ^2^ = 0.3518 with Yates correction, n.s.) but they significantly differ from L2ers (χ^2^ = 7.7651 with Yates correction, significant at *p* < 0.05; 0.01); L2ers significantly differ from Bilinguals (χ^2^ = 9.2427 with Yates correction, significant at *p* < 0.05; 0.01; 0.005). Results are shown in Figure 2.

### Discussion

Results clearly reveal that L2ers use significantly more overt pronouns than Natives and Bilinguals, while Bilinguals from birth behave like Natives in this respect. A significant difference between L2ers on one side and Natives and Bilinguals on the other is observed only with respect to overt pronouns (considered individually or collapsed with ‘other’).

Given that no effect related to cross-linguistic influence can be called into question in this respect for our experimental subjects (as revealed by Study 1), and that Bilinguals and L2ers have a comparable level of proficiency in Italian as attested, the relevant factor that Study 2 singles out is age of onset of exposure to Italian.^24^

Study 2 thus reveals first of all that over-use of overt subject pronouns also occurs in the absence of cross-linguistic influence. Furthermore, Study 2 reveals that it occurs only in a specific group of near-natives: i.e., only in those who have started to acquire the language in question after puberty. A further confirmation of this result is given in the following section.^25^

### Extension

In order to be sure that the results were not a by-product of a ‘stylistic choice’ made by these specific speakers, we compared L2ers productions in Italian with their productions in Greek. If the difference is maintained, it cannot be due to a personal stylistic choice of those speakers, otherwise we should find it also in their Greek productions. As we have shown in the section “The Near-Nativeness Level of the Subjects,” Greek is these subjects’ L1, and, despite their residence in Italy, they have preserved a native level of proficiency in this language (mean value 9.73, range 9.56–9.92). With the same procedure described for Study 1, we collected the materials and derived the Reference Total, consisting of 362 sentences (Supplementary Table 5).

Again, *pro* is the preferred anaphoric device (65.46%) followed by lexical DPs (27.34%), overt pronouns (4.69%) and ‘other’ (2.48%).

**FIGURE 3:**
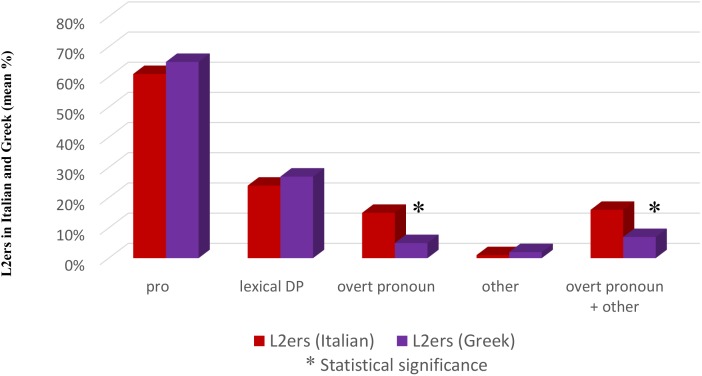
Subject anaphoric devices in L2ers: Italian and Greek.

When we compared the L2ers productions in Greek with their productions in Italian, we found that there are no significant differences with regard to the implementation of *pro* (χ^2^ = 1.4176, n.s.), lexical DPs (χ^2^ = 1.1405, n.s.) and ‘other’ (χ^2^ = 0.7466 with Yates correction, n.s.). There is, however, a significant difference for overt pronouns: these are attested to a significantly higher extent in Italian than in Greek (χ^2^ = 16.8345 with Yates correction, significant at *p* < 0.05; 0.01; 0.005). This significant difference is maintained when overt pronouns and ‘other’ are collapsed (χ^2^ = 10.4534 with Yates correction, significant at *p* < 0.05; 0.01; 0.005). This is shown in Figure 3.

**FIGURE 4:**
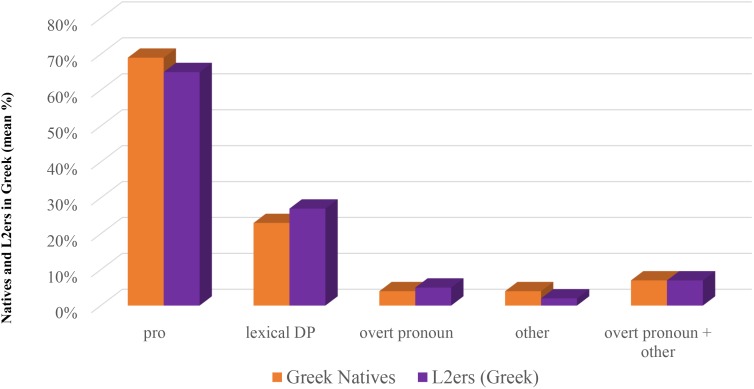
Subject anaphoric devices in Greek: Natives and L2ers.

As a final point, we compared the Greek productions of the L2ers with the Greek productions of the Greek native speakers of Study 1. No significant differences are attested: *pro* (χ^2^ = 1.4095, n.s.), lexical DP (χ^2^ = 1.9132, n.s.), ‘other’ (χ^2^ = 0.6661 with Yates correction, n.s.), overt pronoun (χ^2^ = 0.2495 with Yates correction, n.s.), overt pronouns and ‘other’ (χ^2^ = 0.0010 with Yates correction, n.s.). L2ers over-use overt pronouns in their L2 only, while in their L1 their productions do not differ from those of other native speakers. This is shown in Figure 4.

### Interim Conclusion

In the Introduction, we have briefly reviewed a number of studies that highlighted the role of cross-linguistic influence in determining over-use and over-acceptance of overt subject pronouns in co-reference with a topical antecedent in adult attrited speakers (Tsimpli et al., 2004), adult late acquirers (Sorace and Filiaci, 2006; Belletti et al., 2007) simultaneous bilingual children of a null and a non-null subject language (Serratrice et al., 2004; Sorace et al., 2009, a.o.). As these studies reveal, cross-linguistic influence seems to spread over different populations of bilingual speakers, although in bilingual children developmental factors can be assumed to co-occur in determining its effects, as the results in Sorace et al. (2009) show. Particularly revealing in this respect are the differences between younger and older bilingual children (with the former choosing more overt pronouns), and those concerning monolingual children and monolingual adults (again, with the former choosing more overt pronouns). This fact, together with the observed directionality of cross-linguistic influence (from the non-null subject language to the null-subject language, but not the reverse) suggests that overt pronouns are somehow simpler than null ones. The results of Sorace et al. (2009) together with those of Filiaci et al. (2013) suggest on one side that not all null-subject languages are alike with respect to the division of labor between null and overt subject pronouns, and that cross-linguistic influence may occur also in bilinguals of two null-subject languages (as highlighted by Bini’s (1993) data as well).

Greek and Italian, as Study 1 reveals, are two null subject languages for which no significant quantitative differences are observed in the use of null subject pronouns, overt subject pronouns and subject lexical DPs, so that the results of Study 2 are not an effect of cross-linguistic influence. Here, we can see what cross-linguistic influence seems to obscure, i.e., a difference among different populations of near-natives, which singles out L2ers from bilinguals from birth. Another fact that Study 2 reveals is that, whatever the reason, on which we will not speculate in this work, overt pronouns appear simpler not only for children (as revealed by some of the studies quoted above) but also for adults, when age of onset of exposure to the language in question is rather late.^26^

Absence of cross-linguistic influence has proved thus to offer a fruitful opportunity to study the role of other factors (e.g., age of onset of exposure): with this in mind, we move to Study 3.

## Study 3: the Role of Dominance: Comparing Two Groups of Bilinguals

The results of Study 2 suggest that age of onset of exposure to Italian is a relevant factor in determining the over-use of overt pronouns in near-natives of Italian in the absence of effects related to cross-linguistic influence. Note that the two groups were comparable, despite smaller, non-significant differences, as to the level of proficiency: they were both near-natives, and our aim is to compare natives and near-natives.^27^

Level of proficiency, however, is not the only factor characterizing dominance, and if we want to study the role of dominance in near-natives, other factors have to be taken into consideration.^28^

In order to verify the role of dominance, we decided to compare two different groups of bilinguals: the bilinguals of Study 2, who were living in Greece (Bilinguals in Greece) and a group of bilinguals living in Italy (Bilinguals in Italy). Besides small, non-significant, differences concerning proficiency, the two groups differ in the dimension that concerns the language of the environment (or ‘predominant’ language, see Silva-Corvalán and Treffers-Daller, 2016:3).^29^ Another relevant difference between the two groups concerns use: while Bilinguals in Greece use both Greek and Italian in everyday life, Bilinguals in Italy only use Italian in everyday life, reserving Greek basically for contacts with their family in Greece.

We first compared the Greek of these two groups, and then their Italian. Finally, an interesting comparison is a within-group comparison: the Greek vs. Italian of Bilinguals in Italy as well as the Greek vs. Italian of Bilinguals in Greece.

### Subjects

20 subjects participated in Study 3: the group of 10 Bilinguals living in Greece who participated in Study 2 (Bilinguals in Greece), and a group of 10 bilinguals living in Italy (henceforth Bilinguals in Italy). Bilinguals in Greece have already been described in Study 2. Bilinguals in Italy (4 male; 6 female) had a mean age of 22 (range 19–30). They had all been exposed to both languages since birth, with one parent native speaker of Greek and one parent native speaker of Italian. They grew up mostly in Greece (where they had all attended the Italian State School of Athens) and then they moved to Italy. Their residence in Italy was 6 years on average at the time of testing. As for education, 7 had a high school degree (and were attending university in Italy) and 3 had a university degree (taken in Italy). They were tested in Italy.

### Materials and Methods

#### Ethical Considerations

The same ethical considerations holding for Study 1 (see section “Ethical Considerations”) and Study 2 (see section “Ethical Considerations”) hold here as well.

#### Procedure

The procedure employed to collect the data is the same described for Study 1 and Study 2, the only difference being that Bilinguals in Italy first used Greek and then Italian to tell the story. The procedure to analyze data (sentence typing, derivation of the Reference Total, determination of the subjects’ near-nativeness value) is the same described for Study 1 and Study 2, as well.

### Results^30^

The Reference Total concerning the Greek of Bilinguals in Italy consists of 251 sentences, while for their Italian of 234 sentences. The Reference Total of the Greek of Bilinguals in Greece consists of 267 sentences, while that of their Italian of 241 sentences as described in Study 2.

As mentioned, we will first perform a between-group comparison, initially comparing the Greek of the two groups, then their Italian. We will then proceed to a within-group comparison, first on Bilinguals in Italy, then on Bilinguals in Greece.

#### Bilinguals in Italy vs. Bilinguals in Greece

##### Bilinguals in Italy vs. Bilinguals in Greece: Greek

**FIGURE 5:**
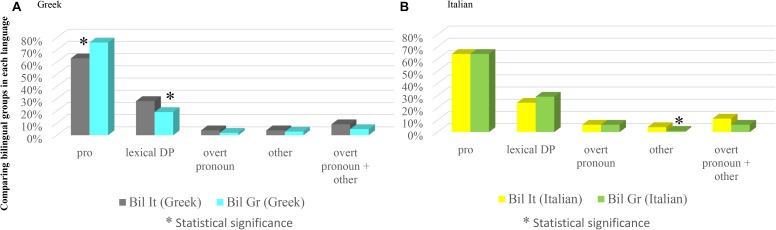
Comparing bilingual groups in each language.

In both groups *pro* is the anaphoric device employed most (63.34% in Bilinguals in Italy; 76.02% in Bilinguals in Greece), followed by lexical DPs (27.88% Bilinguals in Italy; 19.10% Bilinguals in Greece), overt pronouns (4.38% Bilinguals in Italy; 2.24% Bilinguals in Greece) and ‘other’ (4.38% Bilinguals in Italy; 2.62% Bilinguals in Greece). When we compare the percentage rates, we do not find any significant difference concerning overt pronouns (χ^2^ = 1.2466 with Yates correction, n.s.) or ‘other’ (χ^2^ = 0.7285 with Yates correction, n.s.).^31^ The employment of lexical DPs instead differs significantly (χ^2^ = 5.5802, significant at *p* < 0.05), as well as use of *pro*, where the difference is highly significant (χ^2^ = 9.8889, significant at *p* < 0.05; 0.01; 0.005). Bilinguals in Italy use significantly less *pro* and significantly more lexical DPs when compared to Bilinguals in Greece. Results are shown in Figure 5A.

##### Bilinguals in Italy vs. Bilinguals in Greece: Italian

In Italian *pro* is the mostly employed anaphoric device in both groups, too (64.52% Bilinguals in Italy; 63.90% Bilinguals in Greece), followed by lexical DPs (24.35% Bilinguals in Italy; 29.46% Bilinguals in Greece), overt pronouns (6.83% Bilinguals in Italy; 5.80% Bilinguals in Greece) and ‘other’ (4.27% Bilinguals in Italy; 0.82% Bilinguals in Greece). When we turn to the comparisons, we do not find any significant difference as far as overt pronouns are concerned (χ^2^ = 0.0740 with Yates correction, n.s.), but we find a significant difference with respect to ‘other’ (χ^2^ = 4.4045 with Yates correction, significant at *p* < 0.05). The difference doesn’t reach significance when overt pronouns and ‘other’ are collapsed (χ^2^ = 2.4172 with Yates correction, n.s.). We do not find any significant difference with respect to *pro* (χ^2^ = 0.0205, n.s.) or lexical DPs (χ^2^ = 1.5696, n.s.). Results are shown in Figure 5B.

##### Interim discussion

As Figure 5 shows, the Italian of these two groups of speakers is quite uniform, with the exception of a significant difference concerning ‘other,’ more employed by Bilinguals in Italy.

There are indeed some interesting differences concerning the Greek of these two groups of speakers, in that, compared to Bilinguals in Greece, Bilinguals in Italy use significantly less *pro* and significantly more lexical DPs. This could *prima facie* suggest that, although dominance does not affect the productions of overt pronouns, it has some effects in the choice of referring expressions, in that *pro* is less used by those speakers who don’t use this language in everyday life. This conclusion, however, needs further confirmation, since it could be rather the group which uses both languages in everyday life, i.e., Bilinguals in Greece, the one who manifests a peculiarity.

#### Within-Group Comparison

##### Bilinguals in Italy: Greek vs. Italian

**FIGURE 6:**
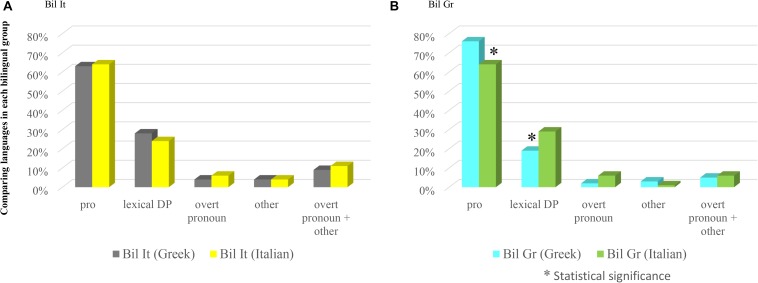
Comparing languages in each bilingual group.

The within-group comparison concerning the Greek and the Italian of Bilinguals in Italy shows that *pro* is the most employed anaphoric device in both languages (63.34% in Greek; 64.52% in Italian) followed by lexical DPs (27.88% in Greek; 24.35% in Italian), overt pronouns (4.38% in Greek; 6.83% in Italian) and other (4.38% in Greek; 4.27% in Italian). The comparison reveals no significant differences with respect to *pro* (χ^2^ = 0.0735, n.s.), lexical DP (χ^2^ = 0.7805, n.s.), overt pronouns (χ^2^ = 0.9608, n.s.), ‘other’ (χ^2^ = 0.0270, n.s.), nor when collapsing overt pronouns and ‘other’ (χ^2^ = 0.5076, n.s.). Results are shown in Figure 6A.

##### Bilinguals in Greece: Greek vs. Italian

The within-group comparison concerning the Greek and the Italian of Bilinguals in Greece shows that *pro* is the most employed anaphoric device in both languages (76.02% in Greek; 63.90% in Italian), followed by lexical DPs (19.10% in Greek; 29.46% in Italian), overt pronouns (2.24% in Greek; 5.80% in Italian), and ‘other’ (2.62% in Greek; 0.82% in Italian). The comparison reveals, however, some significant differences: this is so in the case of *pro* (χ^2^ = 8.9215, significant at *p* < 0.05; 0.01; 0.005) and of lexical DPs (χ^2^ = 7.4494, significant at *p* < 0.05; 0.01). The use of overt pronouns instead does not reveal significant differences in the two languages (χ^2^ = 3.3596 with Yates correction, n.s.), as well as ‘other’ (χ^2^ = 1.4207 with Yates correction, n.s.) or, as expected, collapsing overt pronouns and ‘other’ (χ^2^ = 0.4451 with Yates correction, n.s.). Results are shown in Figure 6B.

##### Interim discussion

The within-group comparison, shown in Figure 6, reveals an interesting fact: while Bilinguals in Italy make the same choice of referential expressions in the language they daily use (Italian) and in the one they seldom use (Greek), Bilinguals in Greece instead differ significantly: they use significantly more *pro* in Greek than in Italian, conversely using more lexical DPs in Italian than in Greek. In contrast, overt pronouns are used to a comparable extent in the two languages.

As we have seen in Study 1, however, in Italian we did not find significant differences between Bilinguals in Greece and Native speakers of Italian, neither for overt pronouns, nor *pro*, nor lexical DPs. There are therefore strong reasons to believe that the difference concerns rather their predominant language, i.e., Greek.

### Extensions and Final Discussion

**FIGURE 7:**
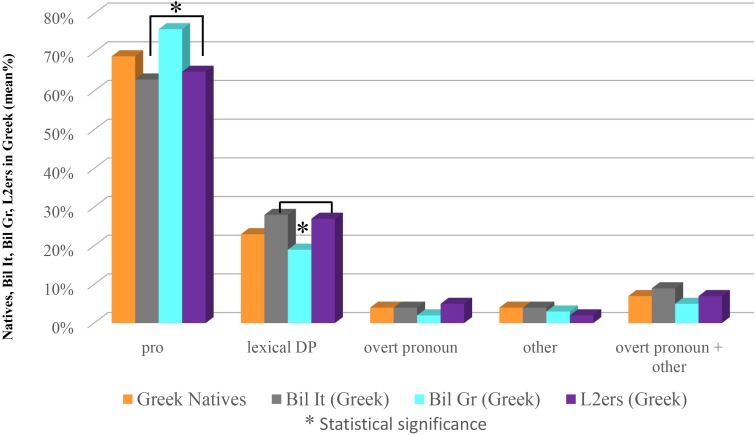
Subject anaphoric devices in Greek: all groups.

At this point, in order to have a clearer picture, we will compare the Greek of all groups discussed in this paper (Natives, Bilinguals in Greece, L2ers, Bilinguals in Italy) as well as their Italian. Let’s start with Greek. As for *pro*, Bilinguals in Greece significantly differ from Bilinguals in Italy (as shown in the section ‘Bilinguals in Italy vs. Bilinguals in Greece: Greek’) and from L2ers (χ^2^ = 8.1529, significant at *p* < 0.05; 0.01) though not from Natives (χ^2^ = 3.6719, n.s.). As for lexical DPs, Bilinguals in Greece again, besides the significant difference with respect to Bilinguals in Italy singled out in the section ‘Bilinguals in Italy vs. Bilinguals in Greece: Greek’) show a significant difference also with respect to L2ers (χ^2^ = 5.7548, significant at *p* < 0.5), though not with respect to Natives (χ^2^ = 1.6077, n.s.). We didn’t find any other significant difference in this comparison: for *pro*, Bilinguals in Italy vs. Natives (χ^2^ = 2.6737, n.s.), Bilinguals in Italy vs. L2ers (χ^2^ = 0.2921, n.s.); for lexical DPs, Bilinguals in Italy vs. Natives (χ^2^ = 1.9631, n.s.), Bilinguals in Italy vs. L2ers (χ^2^ = 0.0217, n.s.); for overt pronouns, Bilinguals in Italy vs. Natives (χ^2^ = 0.0458 with Yates correction, n.s.), Bilinguals in Italy vs. L2ers (χ^2^ = 0.0002 with Yates correction, n.s.), Bilinguals in Greece vs. Natives (χ^2^ = 0.7838 with Yates correction, n.s.), Bilinguals in Greece vs. L2ers (χ^2^ = 1.9670 with Yates correction, n.s.); for ‘other,’ Bilinguals in Italy vs. Natives (χ^2^ = 0.0458 with Yates correction, n.s.), Bilinguals in Italy vs. L2ers (χ^2^ = 1.1414 with Yates correction, n.s.), Bilinguals in Greece vs. Natives (χ^2^ = 0.3559 with Yates correction, n.s.), Bilinguals in Greece vs. L2ers (χ^2^ = 0.0223 with Yates correction, n.s.); for overt pronoun + ‘other,’ Bilinguals in Italy vs. Natives (χ^2^ = 0.2065 with Yates correction, n.s.), Bilinguals in Italy vs. L2ers (χ^2^ = 0.3185 with Yates correction, n.s.), Bilinguals in Greece vs. Natives (χ^2^ = 1.4884 with Yates correction, n.s.), Bilinguals in Greece vs. L2ers (χ^2^ = 1.0442 with Yates correction, n.s.). Results are shown in Figure 7.

**FIGURE 8:**
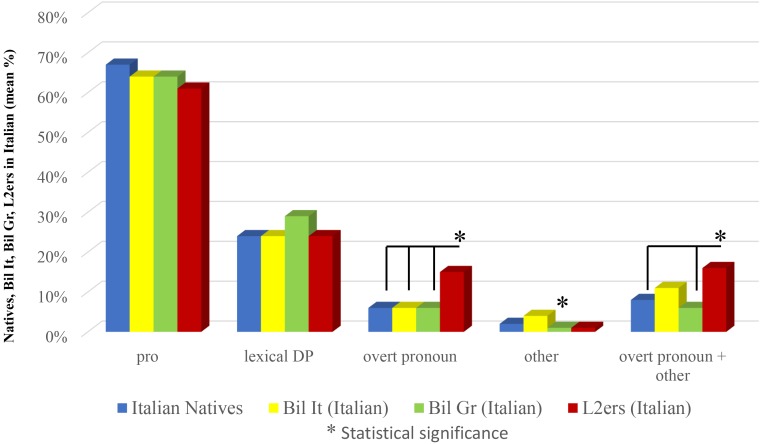
Subject anaphoric devices in Italian: all groups.

As far as Italian is concerned results show that L2ers significantly differ in the use of overt pronouns not only with respect to Natives and Bilinguals in Greece (as shown in Study 2) but also with respect to Bilinguals in Italy (χ^2^ = 6.6599 with Yates correction, significant at *p* < 0.05; 0.01). This significance with respect to Bilinguals in Italy is lost when overt pronouns are collapsed with ‘other’ (χ^2^ = 1.8134 with Yates correction, n.s.). As we have seen in the section “Bilinguals in Italy vs. Bilinguals in Greece: Italian,” Bilinguals in Italy use significantly more ‘other’ than Bilinguals in Greece. It is not so when we compare Bilinguals in Italy to L2ers (χ^2^ = 3.4052 with Yates correction, n.s.) or to Natives (χ^2^ = 1.7991 with Yates correction, n.s.). We didn’t find any other significant difference in this comparison: for *pro*, Bilinguals in Italy vs. Natives (χ^2^ = 0.4588, n.s.), Bilinguals in Italy vs. L2ers (χ^2^ = 0.7310, n.s.); for lexical DPs, Bilinguals in Italy vs. Natives (χ^2^ = 0.0004, n.s.), Bilinguals in Italy vs. L2ers (χ^2^ = 0.0461, n.s.); for overt pronoun, Bilinguals in Italy vs. Natives (χ^2^ = 0.0208 with Yates correction, n.s.); for overt pronoun + ‘other,’ Bilinguals in Italy vs. Natives (χ^2^ = 1.0759 with Yates correction, n.s.). Results are shown in Figure 8.

The comparisons shown in Figure 7, concerning Greek, single out that Bilinguals in Greece use significantly more *pro*s, and significantly less lexical DPs, when compared to both Bilinguals in Italy and L2ers, though not when compared to Natives. The fact that the difference is not restricted to a single group, together with the data described in the section “Within-Group Comparison” allows us to argue that it is precisely this group of speakers that is doing something peculiar, and that is doing so in the predominant language. As we can see in Figure 1 (which pertains to Study 1) native speakers of Greek use more *pro*s and less lexical DPs than Italian natives. This difference, as we noted, is far from significant, however: it has led us to assume that Italian and Greek are very similar with respect to the choice of anaphoric devices. What these bilinguals do, we argue, is amplifying this little difference, modifying their choices in the predominant language. Similar facts have been noted in situations of language contact (see e.g., Scala, 2018 and the references quoted there), where two languages appear more divergent when they are in contact than when they are spoken in non-contact areas, and have been considered therefore a driving factor of language change. As we said in the section “Materials and Methods,” Bilinguals in Greece use Italian (as well as Greek) on a regular basis (differently from Bilinguals in Italy, as well as from L2ers, who use Greek basically just for contacts with their family in Greece). Bilinguals in Greece either attend the Italian State School of Athens or use Italian for their work, and they live in Greece. They are the only group, among our experimental subjects, who employs the two languages in everyday life. Amplifying the differences in the two languages helps these bilingual speakers keeping the two languages separate.

Interestingly, the modification does not involve overt pronouns, but lexical DPs as well as null pronouns. This suggests that overt pronouns are a really marked option, questioning accessibility marking scales such as those in Ariel (1990, 2001) which place overt pronouns near to null ones.

The comparisons shown in Figure 8, concerning Italian, confirm the results of Study 2 (over-use of subject overt pronouns by L2ers) and extend their validity with respect to Bilinguals in Italy (though significance is lost when overt pronouns are collapsed with ‘other’). They also highlight that the significant over-use of ‘other’ by Bilinguals in Italy with respect to Bilinguals in Greece is restricted to this case, hence no reliable conclusions can be drawn in this respect.

## Conclusion

In Study 1, we have presented evidence that native speakers of Greek and of Italian do not differ significantly in the choice of subject anaphoric devices, at least as far as production is concerned: *pro* is overwhelmingly the most attested device, followed by lexical DPs, while overt pronouns are very few in both groups. Italian-Greek is therefore a suitable language combination if we want to study bilinguals’ choices in this respect, since effects related to cross-linguistic influence are absent. This does not mean, of course, that we want to deny, in general, the effects of cross-linguistic influence on the choice of anaphoric devices in bilinguals, since this is clearly demonstrated by several studies. Absence of cross-linguistic influence, however, allows the discovery of other factors playing a role in the issue at stake.

In Study 2, we have compared the productions in Italian of a group of native speakers and two groups of near-natives: a group of bilinguals from birth (Bilinguals in Greece) and a group with post-puberty age of onset of exposure to Italian (L2ers). We have given evidence that over-use of overt subject pronouns in near-natives of Italian takes place when effects related to cross-linguistic influence are absent, singling out that this holds for a specific population of near-natives: those with age of initial exposure to the language in question after puberty. Tsimpli (2014) argues that phenomena which are acquired late (such as pragmatically conditioned aspects of pronominal use) do not cause pronounced differences among bilinguals differing for age of initial exposure. Our study suggests that this claim is valid for pre-puberty but not for post-puberty age of onset of exposure.

As a reviewer wisely observes, the two groups of near-natives in Study 2 do not differ only with respect to age of onset of exposure to Italian, but also with respect to language of the environment: while L2ers live in Italy, Bilinguals in Greece live in Greece. The comparison between L2ers and another group of bilinguals from birth (the Bilinguals in Italy of Study 3) with the same language of the environment as the L2ers (Italian) confirms, however, the very same result: L2ers resort to overt pronouns significantly more than native speakers and bilinguals from birth, as shown in Figure 8.

The language of the environment (or ‘majority language,’ or ‘predominant language’), one of the variables characterizing dominance, does not seem to have an effect on the choice of overt pronouns, as confirmed by Study 3.

This variable, however, combined with regular use of the two languages, has indeed an effect in the choice of anaphoric devices such as *pro* and lexical DPs, though in a direction we did not expect: it is in the predominant language, rather than in the non-predominant one, that differences between natives and bilinguals have been observed. We have interpreted these differences as stemming from the bilinguals’ need to keep the two languages they daily use as distant as possible. Interestingly, these differences do not involve overt pronouns, but concern a wider use of null pronouns which charges lexical DPs. This suggests that overt pronouns are a marked option, questioning accessibility marking scales such as those in Ariel (1990, 2001) which place overt pronouns near to null ones. As a reviewer suggests, the significantly higher use of *pro* in Greek by Bilinguals in Greece might also reflect an underlying property of Greek *pro*, which, according to some authors appears to be compatible with salient/subject antecedent but also with non-salient/ object antecedent (Dimitriadis, 1996; Torregrossa et al., 2015). At a first analysis, our data are not very clear in this respect, and we have to leave this issue for future research.

A final note concerns the small-scale nature of our corpora, which has proven particularly limiting in the case of overt pronouns (which are seldom produced by our subjects) preventing a serious qualitative analysis of the contexts in which they occur in natives, bilinguals and L2ers. Another issue which we leave for future research is thus an inquiry with a wider range of data, collected with the help of different tasks, as a reviewer suggests.

## Author Contributions

EDD developed the rationale of the study, the study concept and design, and wrote the manuscript. IB contributed in data collection. EDD and IB contributed in data analysis and its interpretation. Both authors critically read the manuscript providing comments that helped to improve its final version, and approved the final version for submission.

## Conflict of Interest Statement

The authors declare that the research was conducted in the absence of any commercial or financial relationships that could be construed as a potential conflict of interest.

## Footnotes

1 Greek third person personal pronouns disappeared around the 5th–6th century B.C. (Panagiotidis, 2000) and were substituted by demonstratives, as *aftos* in (3.c; this one) or *ekinos* (that one), with an anaphoric function. These demonstrative pronouns can also have an inanimate antecedent, contrary to Italian overt pronouns. In Italian, demonstrative pronouns can also be used with anaphoric function, with inanimate referents only or, with a pejorative flavor, in sub-standard varieties with animate/human referents. (3.a) shows another difference between Greek and Italian, in that Greek freely allows post-verbal subjects (Roussou and Tsimpli, 2006), and VSO, while in Italian the post-verbal position of subjects is restricted to new-information focus subjects and VSO is impossible (Belletti, 2001, 2004). Post-verbal subjects and VSO are also possible in Spanish, although Greek and Spanish partly differ, word order being more flexible in Greek than in Spanish, since VSO is allowed by different mechanisms in the two languages (Roussou and Tsimpli, 2006).

2 The null pronoun, for instance, binds the anaphor *se stesso* in (i.c) as the lexical DP *Gianni* does in (i.a). Contrary to pronouns (*lo* in i.b), anaphors must be bound within the clause containing them:

(i) a. Paolo_i_ ha detto [che [Gianni_j_ proteggerà se stesso_j_]]
  P. said that G. will protect himself
b. Paolo_i_ ha detto [che [Gianni_j_ lo_^∗^J_ proteggerà]]
  P. said that G. him will protect
c. Paolo_i_ ha detto [che [*pro*_i_ proteggerà se stesso_i]_]
  P. said that he will protect himself

3 Experimental findings by Carminati (2002) suggest indeed that *pro*, at least in intra-sentential anaphora, looks for an antecedent in Spec, IP. Subjects of predication share properties with topics: according to Rizzi (2005, 2018), subjects and topics share an ‘aboutness’ property. According to Lambrecht (1994: 118) topics are ‘the thing which the proposition expressed by the sentence is about.’

4 Do not make your contribution more informative than is required’ [Grice, 1975: 45]. Calabrese (1986: fn. 6) suggests that the Avoid Pronoun Principle (Chomsky, 1981: 65) must be interpreted in a similar vein.

5 A similar claim is made by Chiou (2013) for Greek.

6 Note that (8.b) and (9.b) are possible with a different indexing, i.e., if *lui/aftos* does not co-refer with *Mario*.

7 The study was also concerned with post-verbal subjects, which are possible in null-subject languages but not in non-null subject languages, as originally noted by Rizzi (1982). As noted in footnote 1, Greek and Italian, however, differ in this respect.

8 Results concerning the interpretation of null and overt pronouns are presented in Tsimpli et al. (2004) only for the Italian participants. Experimental sentences were of the kind given in (i.a) and (i.b):

(i) a. L’anziana signora saluta la ragazza quando lei attraversa la strada
  The old woman greets the girl when she is crossing the street
b. Quando lei attraversa la strada, l’anziana signora saluta la ragazza
  When she is crossing the street, the old woman greets the girl

Results were particularly clear in the (i.a) condition. These results concerning the comprehension of overt pronouns are however not matched in the production tasks, such as the Story Telling task, for which, as the authors acknowledge, no significant results were attested for either group (Tsimpli et al., 2004: 267).

9 Experimental materials were very similar to those employed by Tsimpli et al. (2004). Here as well, results were particularly clear in cases like (i.a) of footnote 8.

10 Several studies tackle indeed this issue examining spontaneous productions of bilingual children with a null and a non-null subject language. See, among others, Paradis and Navarro (2003) on a Spanish-English child, Pinto (2006) on two Dutch-Italian children), Hacohen and Schaeffer (2007) on a Hebrew-English child. They all found an over-use of overt subjects in the null-subject language.

11 The authors propose difficulties at integrating different types of information in real time as an explanation for their results. Along the same line, Sorace (2011) suggests that there could also be a difference in the processing resources available for bilingual and monolingual speakers.

12 The Pear Film is a 6-min film without dialogs. It was created at the University of California at Berkeley in 1975 by a group of linguists to collect narration data. See Chafe (1980) for a first report of this research. Years later, data collected through the Pear Film have become part of the experimental material in works dedicated to the study of pronoun production and resolution in bilingual contexts, such as Tsimpli et al. (2004) and Belletti et al. (2007).

13 Greek doesn’t have infinitives, but rather embedded sentences introduced by *na* or *ke* complementizers whose verbs are inflected. Verbs in the matrix clause that embed a *na* or *ke* complement clause are perception, knowledge aspectual and modal verbs (for a complete list see e.g., Ingria, 2005; Spyropoulos, 2007). For these cases, there is disagreement in the relevant literature as to the kind of subjects inside these clausal types (see e.g., Philippaki-Warburton and Catsimali, 1999; Alexiadou and Anagnostopoulou, 2002; Spyropoulos, 2007 among others, and the references quoted there) but all analyses agree on the fact that inside these complement clauses the subject (as well as tense) is dependent on the subject of the matrix clause and can never be overt. *na* clauses can also occur as independent clauses and in this case they are considered subjunctive clauses (hence with an independent subject, which can be null or overt). Matrix clauses with verbs like *elpizo* (hope), *perimeno* (wait/expect), *pistevo* (believe) embed *na* clauses whose subject (and tense) is independent from the one of the matrix clause (Spyropoulos, 2007).

14 Greek has two kinds of existential sentences, those involving the verb *ine* (be-3sg/pl) and those involving the verb *echi* (have-3sg), whose subject can never be overt. Italian existential sentences contain the locative clitic *ci* and the verb *essere* (be), and a so called ‘pivot’ which can be definite or indefinite. In the type of existential most attested in our corpus, the one containing an indefinite pivot, the (expletive) subject is assumed to be *ci*.

15 Some of the sentences with a ‘narrator’ subject were indeed stock phrases such as Gr. *xero go* (I don’t know, lit. know I) or It. *diciamo* (let’s say).

16 Results are summarized in Supplementary Table 1 (Italian Natives) and Supplementary Table 2 (Greek Natives), where the Reference Total of the sentences for the two groups is shown, together with the indication of the clausal type and of the occurrences and percentages of the kind of referring expression employed.

17 Given the small-scale nature of the data discussed in this study, we chose the χ^2^-test as a suitable non-parametric procedure to analyze our data. Group responses are indeed quite representative of individual ones, as revealed by *a* ≤ 0.5 coefficient of variation in responses for *pro*, lexical DPs and overt pronouns in Greek natives and for *pro* and lexical DPs in Italian natives. The latter holds for all the experimental groups discussed in the present work.

18 As noted in footnote 1, Greek allows post-verbal subjects more than Italian. Our data support this fact in that post-verbal subjects in the Reference Total of the Greek natives are much more widespread (50.35%) than in the Reference Total of the Italian natives (21.42%). The difference is highly significant (χ^2^ = 22.6082 with Yates correction, significant at *p* < 0.05, 0.01, and 0.005).

19 See section “The Near-Nativeness Level of the Subjects” for more information concerning their level of proficiency in the two languages.

20 Two of the Italian judges (2 male; 1 female, aged 25–29, living in Italy) were teachers of Italian as an L2, and another was working for an organization for immigrants.

21 The Greek judges (1 male; 2 female, aged 25–31, living in Greece) were teachers of Greek as an L2.

22 Supplementary Table 9 reports the mean value of (near-) nativeness for each group of experimental subjects participating in Study 1, Study 2 and Study 3.

23 Supplementary Table 3 reports the Reference Total concerning Bilinguals in Greece, together with the indication of the clausal type and of the occurrences and percentages of the kind of referring expression employed. The same is shown in Supplementary Table 4 for the L2ers. As for Natives, as already presented, the same is shown in Supplementary Table 1.

24 Given the differences between Greek and Italian outlined in footnotes 1 and 18, we could expect cross-linguistic influence from Greek to Italian with respect to post-verbal subjects and the use of demonstratives for our L2ers. This is however not the case: subjects in post-verbal position are 15% in the L2ers Reference Total (even less than in the Italian natives Reference Total) while demonstratives amount to only 10.8% of overt pronominal devices (8.33% in the Italian natives Reference Total).

25 These results are also strengthened, as we shall see, by the findings in Study 3, where the productions of another group of bilinguals from birth (Bilinguals living in Italy) are analyzed. These bilinguals too, do not over-use overt pronouns in either of their languages.

26 A brief examination of the contexts in which overt pronouns occurred reveals that while native speakers and bilinguals use them in topic shift contexts, in L2ers’ productions this is often not the case, especially when more than one Discourse Referent is active at some specific points of the narration. Overt pronouns were however very few in our corpora, and the issue needs to be studied more in depth and with a wider range of data. We therefore leave the issue for future research.

27 L2ers had a mean value of 8.88/10 in Italian and of 9.73/10 in Greek. Bilinguals in Greece had a mean value of 8.98/10 in Italian and of 9.34/10 in Greek. These differences (either within-group or between-group) are non-significant: L2ers Italian vs. Greek: χ^2^ = 0.0174 with Yates correction, n.s.; Bilinguals in Greece: Greek vs. Italian χ^2^ = 0,2662 with Yates correction, n.s.; L2ers vs. Bilinguals in Greece, Italian: χ^2^ = 0.4239, with Yates correction, n.s.; L2ers vs. Bilinguals in Greece, Greek; χ^2^ = 0.4196 with Yates correction, n.s. The data concerning Bilinguals in Greece raise an interesting issue that we leave for future research, since Bilinguals in Greece appear to be near-natives in both Italian and Greek, i.e., they appear as native speakers of none of their two languages.

28 See a.o Birdsong (2014), Montrul (2016), Silva-Corvalán and Treffers-Daller (2016), Treffers-Daller and Korybski (2016) and the references quoted there.

29 Bilinguals in Italy had a mean value in Italian of 9.03 (range 8.69–9.38) and of 8.79 in Greek (range 8.08–9.24). As we noticed for the Bilinguals in Greece, Bilinguals in Italy appear to be native speakers of neither of their languages, too. Another fact worth noting is that their residence in Italy seems to have had a greater effect on their Greek, when compared to L2ers, which still maintain their native level in Greek notwithstanding the years spent in Italy: attrition seems to have a more pervasive effect in bilinguals than in L2ers. The near-nativeness values in each language of this group of experimental subjects is also reported in Supplementary Table 9. As for within-group and between-group statistical significance, we observed the following: Bilinguals in Italy: Greek vs. Italian χ^2^ = 0,2974 with Yates correction, n.s.; Bilinguals in Greece vs. Bilinguals in Italy: Greek χ^2^ = 0,1195 with Yates correction, n.s.; Italian χ^2^ = 0,5036 with Yates correction, n.s.

30 The Reference Total concerning the Greek of Bilinguals in Italy is reported in Supplementary Table 6, while the Reference Total concerning the Italian of Bilinguals in Italy is reported in Supplementary Table 7. Supplementary Table 8 reports the Reference Total concerning the Greek of Bilinguals in Greece, while the Reference Total concerning their Italian is shown in Supplementary Table 3. The tables show the Reference Total of the sentences produced, together with the clausal type and the occurrences and percentages of the kind of referring expression employed.

31 When pronouns and other are collapsed we do not reach significance either, as expected (χ^2^ = 2.5295 with Yates correction, n.s.).
