# Supplementary material for: Age of Onset and Dominance in the Choice of Subject Anaphoric Devices: Comparing Natives and Near-Natives of Two Null-Subject Languages
Source: Front Psychol. 2019 Jan 10;9:2729. doi: 10.3389/fpsyg.2018.02729 (PMC6337714; doi:10.3389/fpsyg.2018.02729)
Supplement: Supplementary file 1 [file Table_1.DOCX]

Supplementary Material

Age of Onset and Dominance in the Choice of Subject Anaphoric Devices: Comparing Natives and Near- natives of two Null- subject Languages

Elisa Di Domenico, Ioli Baroncini

*** Correspondence:** elisa.didomenico@unistrapg.it

**TABLE 1**: Italian Natives: Reference Total

| Italian Natives: Reference Total | | | | | |
| --- | --- | --- | --- | --- | --- |
|  | number of sentences | pro | lexical DP | overt pronoun | other |
| Finite sentences | 362 | 246 | 88 | 21 | 6 |
| Copular sentences | 8 | 3 | 2 | 1 | 2 |
| Relative clauses | 17 | 11 | 4 | 2 | 0 |
| Total | 387 | 260 (67.18%) | 94 (24.28%) | 24  (6.20%) | 8 (2.06%) |

**TABLE 2**: Greek Natives: Reference Total

| Greek Natives: Reference Total | | | | | |
| --- | --- | --- | --- | --- | --- |
|  | number of sentences | pro | lexical DP | overt pronoun | other |
| Finite sentences | 404 | 291 | 87 | 15 | 11 |
| Copular sentences | 22 | 10 | 4 | 2 | 6 |
| Relative clauses | 28 | 14 | 14 | 0 | 0 |
| Clefts | 1 | 1 | 0 | 0 | 0 |
| Total | 454 | 315 (69.38%) | 105 (23.12%) | 17  (3.74%) | 17 (3.74%) |

**TABLE 3**: Bilinguals in Greece (Italian): Reference Total

| Bilinguals in Greece (Italian): Reference Total | | | | | |
| --- | --- | --- | --- | --- | --- |
|  | number of sentences | pro | lexical DP | overt pronoun | other |
| Finite sentences | 220 | 142 | 62 | 14 | 2 |
| Copular sentences | 9 | 6 | 3 | 0 | 0 |
| Relative clauses | 12 | 6 | 6 | 0 | 0 |
| Total | 241 | 154 (63.90%) | 71 (29.46%) | 14  (5.80%) | 2 (0.82%) |

**TABLE 4**: L2ers (Italian): Reference Total

| L2ers (Italian): Reference Total | | | | | |
| --- | --- | --- | --- | --- | --- |
|  | number of sentences | pro | lexical DP | overt pronoun | other |
| Finite sentences | 242 | 147 | 58 | 35 | 2 |
| Copular sentences | 8 | 5 | 1 | 1 | 1 |
| Relative clauses | 5 | 3 | 1 | 1 | 0 |
| Total | 255 | 155 (60.78%) | 60  (23.52%) | 37  (14.50%) | 3  (1.17%) |

**TABLE 5**: L2ers (Greek): Reference Total

| L2ers (Greek): Reference Total | | | | | |
| --- | --- | --- | --- | --- | --- |
|  | number of sentences | pro | lexical DP | overt pronoun | other |
| Finite sentences | 329 | 219 | 86 | 16 | 8 |
| Copular sentences | 10 | 7 | 3 | 0 | 0 |
| Relative clauses | 23 | 11 | 10 | 1 | 1 |
| Total | 362 | 237 (65.46%) | 99 (27.34%) | 17  (4.69%) | 9  (2.48%) |

**TABLE 6**: Bilinguals in Italy (Greek): Reference Total

| Bilinguals in Italy (Greek): Reference Total | | | | | |
| --- | --- | --- | --- | --- | --- |
|  | number of sentences | pro | lexical DP | overt pronoun | other |
| Finite sentences | 226 | 144 | 61 | 10 | 11 |
| Copular sentences | 9 | 6 | 2 | 1 | 0 |
| Relative clauses | 16 | 9 | 7 | 0 | 0 |
| Total | 251 | 159  (63.34%) | 70  (27.88%) | 11  (4.38%) | 11  (4.38%) |

**TABLE 7**: Bilinguals in Italy (Italian): Reference Total

| Bilinguals in Italy (Italian): Reference Total | | | | | |
| --- | --- | --- | --- | --- | --- |
|  | number of sentences | pro | lexical DP | overt pronoun | other |
| Finite sentences | 216 | 137 | 54 | 15 | 10 |
| Copular sentences | 5 | 3 | 1 | 1 | 0 |
| Relative clauses | 13 | 11 | 2 | 0 | 0 |
| Total | 234 | 151  (64.52%) | 57  (24.35%) | 16  (6.83%) | 10  (4.27%) |

**TABLE 8**: Bilinguals in Greece (Greek): Reference Total

| Bilinguals in Greece (Greek): Reference Total | | | | | |
| --- | --- | --- | --- | --- | --- |
|  | number of sentences | pro | lexical DP | overt pronoun | other |
| Finite sentences | 244 | 188 | 47 | 6 | 4 |
| Copular sentences | 9 | 6 | 0 | 0 | 2 |
| Relative clauses | 12 | 8 | 3 | 0 | 1 |
| Clefts | 2 | 1 | 1 | 0 | 0 |
| Total | 267 | 203 (76.02%) | 51  (19.10%) | 6  (2.24%) | 7  (2.62%) |

**TABLE 9**: Mean near-nativeness values in the experimental groups

|  | Italian Natives | Greek Natives | Bilinguals in Greece | | Bilinguals in  Italy | | L2ers | |
| --- | --- | --- | --- | --- | --- | --- | --- | --- |
|  |  |  | Italian | Greek | Italian | Greek | Italian | Greek |
| Mean value | 9.79 | 9.87 | 8.98 | 9.34 | 9.03 | 8.79 | 8.88 | 9.73 |
| Range | 9.64 - 9.96 | 9.64 -9.96 | 8.74 -9.28 | 8.61 -9.80 | 8.69 -9.38 | 8.08 -9.24 | 8.50 -9.33 | 9.56 -9.92 |
